# Atypical Radiological Presentation of a Neonatal Primary Retroperitoneal Teratoma Undergoing Haemorrhagic Evolution: A Case Report

**DOI:** 10.7759/cureus.82217

**Published:** 2025-04-13

**Authors:** Sana Shah Alam, Jeevika Ujjappa

**Affiliations:** 1 Radiodiagnosis, J.J.M. Medical College, Davanagere, IND

**Keywords:** congenital tumor, extragonadal germ cell tumors, haemorrhagic infarction, neonatal abdominal mass, retroperitoneal teratoma

## Abstract

Primary retroperitoneal teratomas are rare extragonadal germ cell tumours that originate from totipotent embryonic cells misplaced during embryogenesis. These tumors are more commonly seen in neonates and infants and may remain asymptomatic until they reach a significant size, leading to compressive symptoms. Here, we present a case of a 15-day-old neonate with progressive abdominal distension since birth and a fever for three days. Prenatal ultrasonographic imaging at 22-23 weeks gestation detected an intra-abdominal cystic lesion, initially suspected to be a duplication or mesenteric cyst. By 29-30 weeks, the lesion showed solid components with possible calcifications, raising suspicion of an intra-abdominal teratoma. Through detailed clinical evaluation, radiographic imaging, and diagnostic modalities, including chest radiography, ultrasound, computed tomography and magnetic resonance imaging, the anatomical features and associated complications were delineated. Surgical excision was performed, and histopathological examination revealed haemorrhagic infarction of a teratoma, confirming the diagnosis. Primary retroperitoneal teratomas in neonates are uncommon and may mimic other congenital cystic or solid abdominal masses. This case underscores the importance of early detection and multidisciplinary management in optimizing outcomes.

## Introduction

Primary retroperitoneal teratomas are a rare group of extragonadal germ cell neoplasms composed of tissues from multiple embryonic germ cell layers arising outside the gonads. They account for only a small fraction (~5%) of retroperitoneal tumors, with a bimodal age distribution (peaking in infancy and early adulthood) and a slight female predilection. In the pediatric population, they are the third most frequent primary retroperitoneal tumor, following neuroblastoma and Wilms tumor [1]. Clinically, these tumors remain asymptomatic until they attain a considerable size, at which point the patients may present with a palpable abdominal mass, abdominal distension, pain, or other compressive symptoms​ [1]. Rarely, they may present with acute complications like rupture or malignant transformation, especially in long-standing cases [2]. Diagnostic evaluation relies on imaging studies like ultrasonography, computed tomography (CT) and magnetic resonance imaging (MRI), which typically reveals a complex heterogeneous mass containing cystic components, fat, and calcifications (such as teeth or bone), features highly suggestive of a teratoma. Serum tumor markers (alpha-fetoprotein, carcinoembryonic antigen, CA-19-9) may be elevated in some cases and can assist in the assessment and monitoring of the tumor [3]. Important differential diagnoses include other retroperitoneal tumors with fat or calcifications (e.g., liposarcoma or myelolipoma) and metastatic germ cell tumors. These neoplasms are notably resistant to chemotherapy and radiation; such adjuvant therapies are reserved for unresectable disease or documented malignant components [4].

## Case presentation

A 15-day-old neonate presented with complaints of abdominal distention since birth, along with a history of fever for the past three days. The mother was 32 years old G2P1L1A0 with routine prenatal scans conducted at 6 and 13 weeks of gestation at an external facility, reported as normal. Subsequent prenatal ultrasound scans were performed at 28-29 weeks, which identified a single live intrauterine gestation corresponding to gestational age, with an intra-abdominal cyst located medial to the stomach bubble. The differential diagnosis at that time included a duplication cyst or mesenteric cyst. A follow-up scan at 34-35 weeks revealed a more complex intra-abdominal cystic lesion with a solid component and possible calcification, raising suspicion for an intra-abdominal teratoma. The previous pregnancy was uneventful. Physical examination of the neonate revealed a firm, non-tender, palpable mass occupying the whole of the abdominal cavity, with the neonate exhibiting normal vital signs.

**Figure 1:**
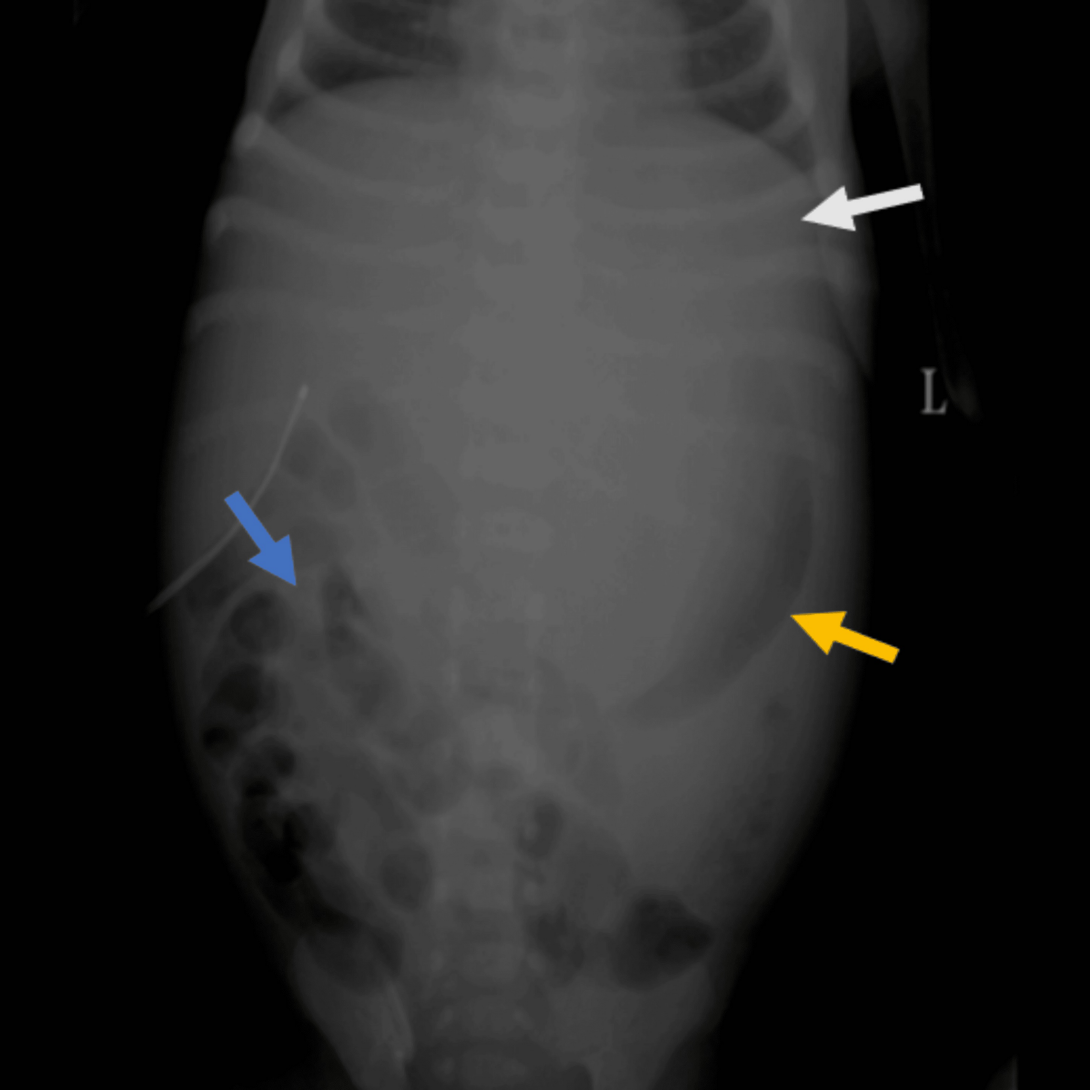
Abdominal radiograph in the posteroanterior view demonstrates haziness in the upper abdomen with non-visualization of the stomach bubble beneath the left hemidiaphragm (white arrow). A crescent-shaped air lucency is identified in the left lumbar region (yellow arrow). The small and large bowel loops appear displaced and compressed inferiorly and laterally into the right iliac fossa and hypogastric region (blue arrow). Loss of the preperitoneal fat stripe is noted.

A preliminary abdominal radiograph (Figure 1) was obtained, which demonstrated diffuse haziness in the upper abdomen with non-visualization of the stomach bubble beneath the left hemidiaphragm. A crescent-shaped air lucency was identified in the left lumbar region. The small and large bowel loops appeared displaced and compressed inferiorly and laterally into the right iliac fossa and hypogastric region, likely secondary to mass effect from a lesion situated in the upper abdomen. The loss of the pre-peritoneal fat stripe is suggestive of possible ascites. No abnormal calcifications were identified within the abdominal cavity. The radiographic features were suggestive of mass effects secondary to a soft tissue density lesion in the upper abdomen.

**Figure 2:**
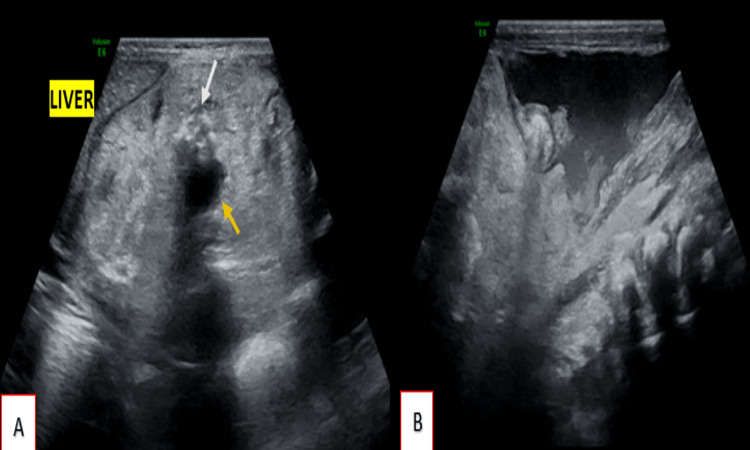
(A) Well-defined, heterogeneous, predominantly hyperechoic solid-cystic mass lesion measuring approximately 5.0 x 6.0 x 5.5 cm in the epigastric region. The lesion is adjacent to the liver and demonstrates multiple hypoechoic cystic areas (yellow arrow) and foci of calcifications (white arrow). The lesion exerts significant compression and superior displacement of both hepatic lobes; however, clear fat planes separating the lesion from the liver are maintained. (B) Free fluid in pelvic cavity showing echogenic debris.

Further imaging studies, including ultrasound, CT and MRI, were performed for definitive characterization. Grayscale ultrasonographic evaluation revealed a well-defined, heterogeneous, predominantly hyperechoic solid-cystic mass lesion (Figure 2), measuring approximately 5.0 x 6.0 x 5.5 cm in the epigastric region, extending inferiorly up to the level of the umbilicus. The lesion was located adjacent to the liver and demonstrated multiple hypoechoic cystic areas with multiple foci of calcification, giving posterior acoustic shadowing. The lesion exerted significant compression and superior displacement of both hepatic lobes; however, clear fat planes separating the lesion from the liver were maintained. Free fluid was identified within the sub-hepatic, peri-hepatic, peri-splenic, pelvic, and general peritoneal spaces. The fluid exhibited echogenic debris with multiple thin internal septations. The mass lesion appeared distinctly separate from the bowel loops.

**Figure 3:**
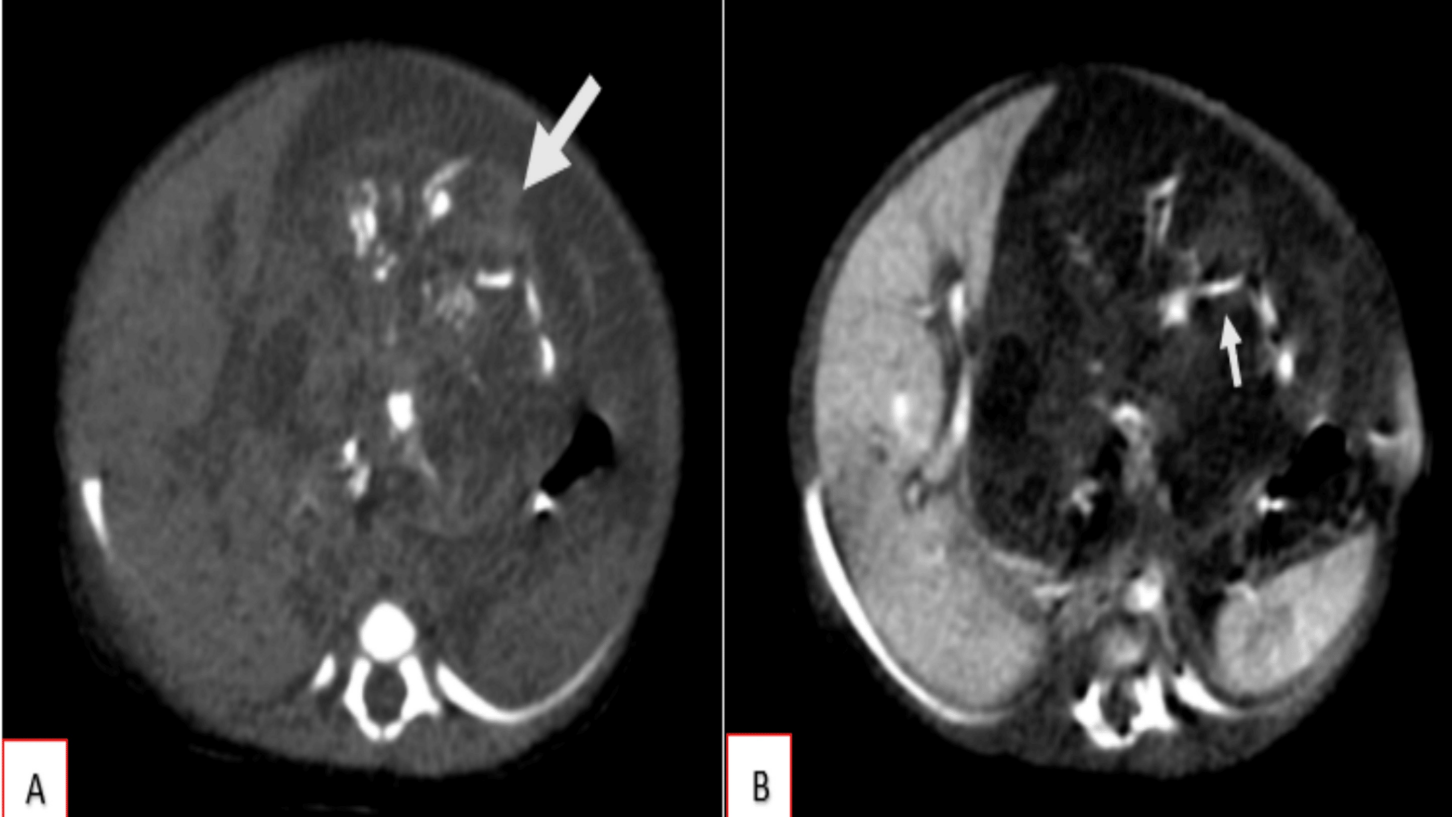
Plain (A) and contrast-enhanced (B) axial sections demonstrate a large, solitary, well-defined, heterogeneously iso- to hypodense solid-cystic mass lesion (white arrow) located along the midline and occupying the epigastric, left hypochondriac, and umbilical regions. The lesion measures approximately 7.2 cm (craniocaudal) × 6.8 cm (anteroposterior) × 6.3 cm (transverse). Within the lesion, multiple foci of linear and curvilinear calcifications are seen centrally (HU: 280) (smaller white arrow). No significant contrast enhancement is noted. Fat planes with adjacent solid organs and bowel loops are maintained.

Serial plain and contrast-enhanced CT scans of the abdomen and pelvis were obtained, which revealed a large, solitary, well-defined, heterogeneously iso-hypodense solid-cystic mass lesion (Figure 3) with attenuation values measuring approximately 38 Hounsfield Units (HU), situated along the midline and occupying the epigastric, left hypochondriac, and umbilical regions. The lesion measured approximately 7.2 cm (craniocaudal) x 6.8 cm (anteroposterior) x 6.3 cm (transverse). Within the lesion, multiple foci of linear and curvilinear calcifications (HU: 280) were identified centrally. No significant contrast enhancement was noted, with pre-contrast attenuation of approximately 50 HU and post-contrast attenuation of 52 HU. There was no evidence of communication with adjacent solid organs or bowel loops. No areas of fatty attenuation are noted within the lesion.

**Figure 4:**
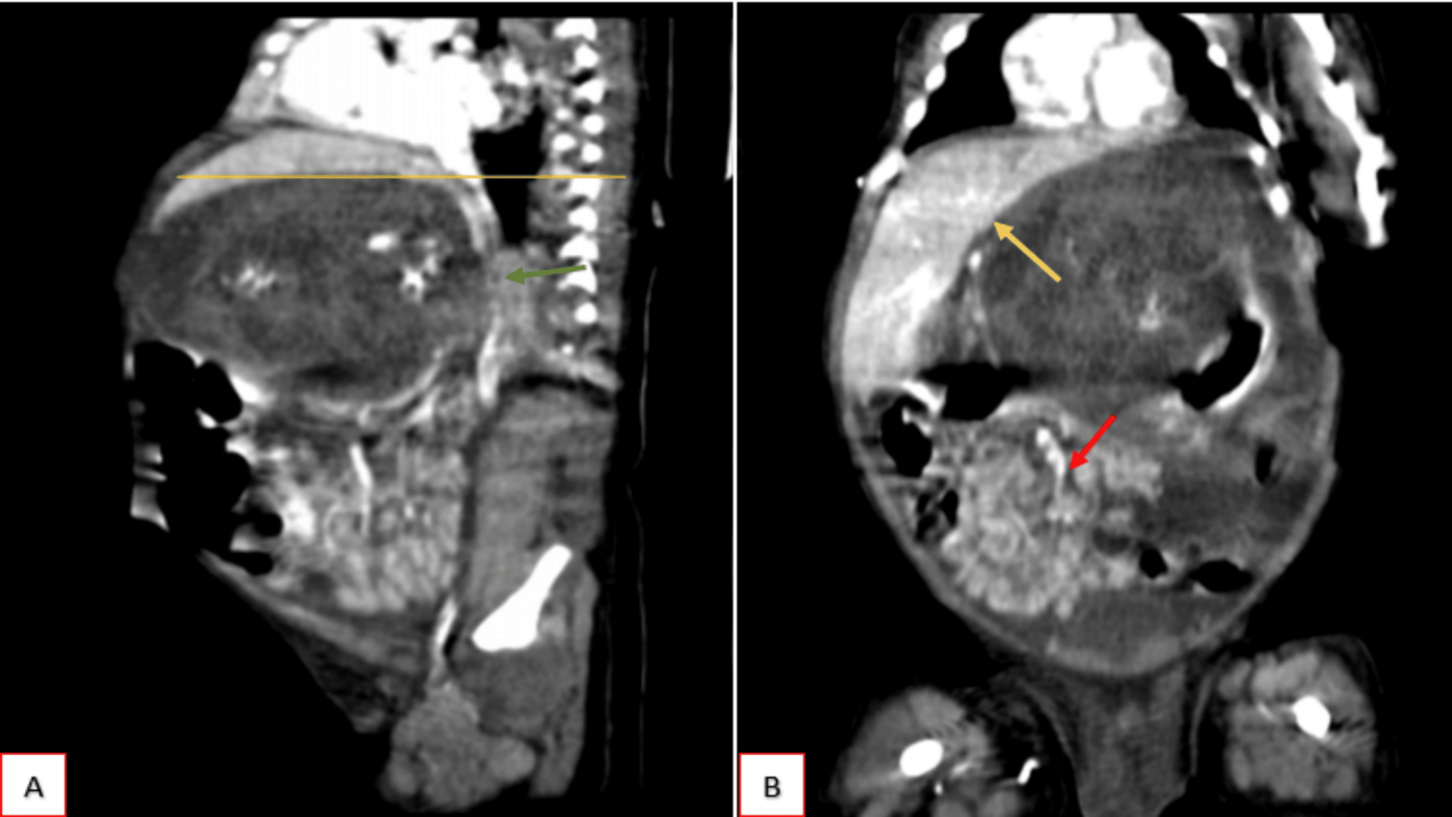
Contrast enhanced CT in sagittal (A) and coronal (B) section shows superiorly the lesion extending up to the superior endplate of the D7 vertebra (yellow line), compressing and laterally displacing the liver, particularly the left lobe, abutting the left hemidiaphragm (yellow arrow). The lesion is extending downwards upto the level of the inferior endplate of the L2 vertebra, displacing bowel loops inferiorly (red arrow). There is posterior compression on the abdominal aorta, celiac trunk, superior mesenteric artery (SMA), and its branches (green arrow), anteriorly compression on the abdominal wall noted.

In contrast to the enhanced sagittal and coronal sections (Figure 4), the lesion extended up to the superior endplate of the D7 vertebra, compressing and laterally displacing the liver, particularly the left lobe, and abutting the left hemidiaphragm; however, fat planes with the liver remained preserved. The lesion extended downwards to the level of the inferior endplate of the L2 vertebra, displacing bowel loops inferiorly. Posteriorly, there was extrinsic compression on the abdominal aorta, celiac trunk, superior mesenteric artery (SMA), and its branches. No filling defects within these vessels were identified. Anteriorly, the lesion compressed the anterior abdominal wall.

**Figure 5:**
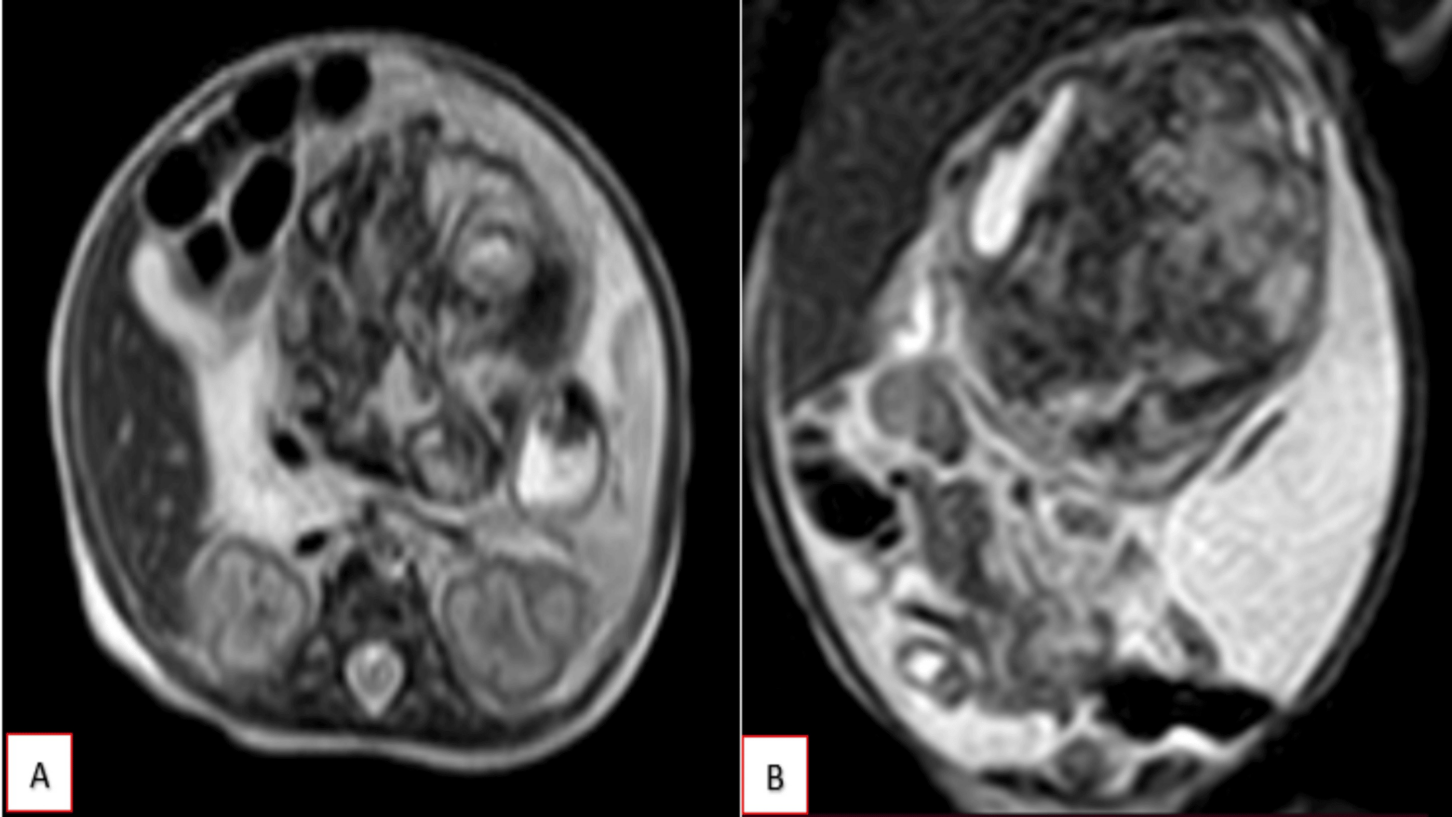
MRI T2-weighted sequence in axial (A) and coronal (B) sections shows a well-defined mass lesion located along the midline. The lesion appears hyperintense on T2-weighted images.

**Figure 6:**
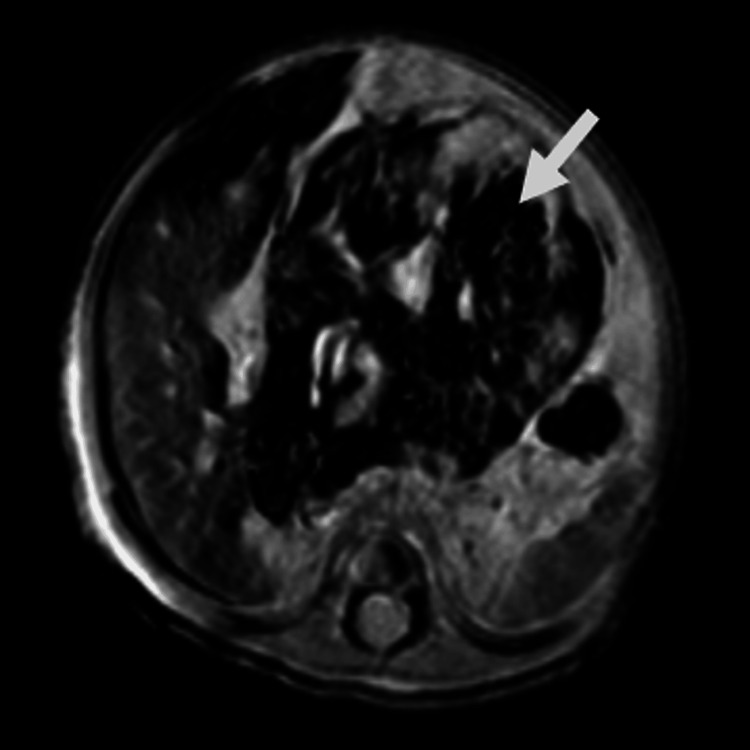
MRI gradient-recalled echo (GRE) sequence in axial section shows multiple areas demonstrating blooming artifacts (white arrow), suggestive of possible calcifications or haemorrhage within the lesion.

A complementary limited MRI study was performed, demonstrating a well-defined mass lesion located along the midline. The lesion appeared hypointense on T1-weighted images and heterogeneously hyperintense on T2-weighted images (Figure 5). Areas demonstrating susceptibility artifacts ("blooming") were noted on gradient-recalled echo (GRE) sequences (Figure 6), indicative of possible calcifications or haemorrhage within the lesion. These imaging characteristics further support the complexity of the lesion and aid in narrowing differential diagnoses.

**Figure 7:**
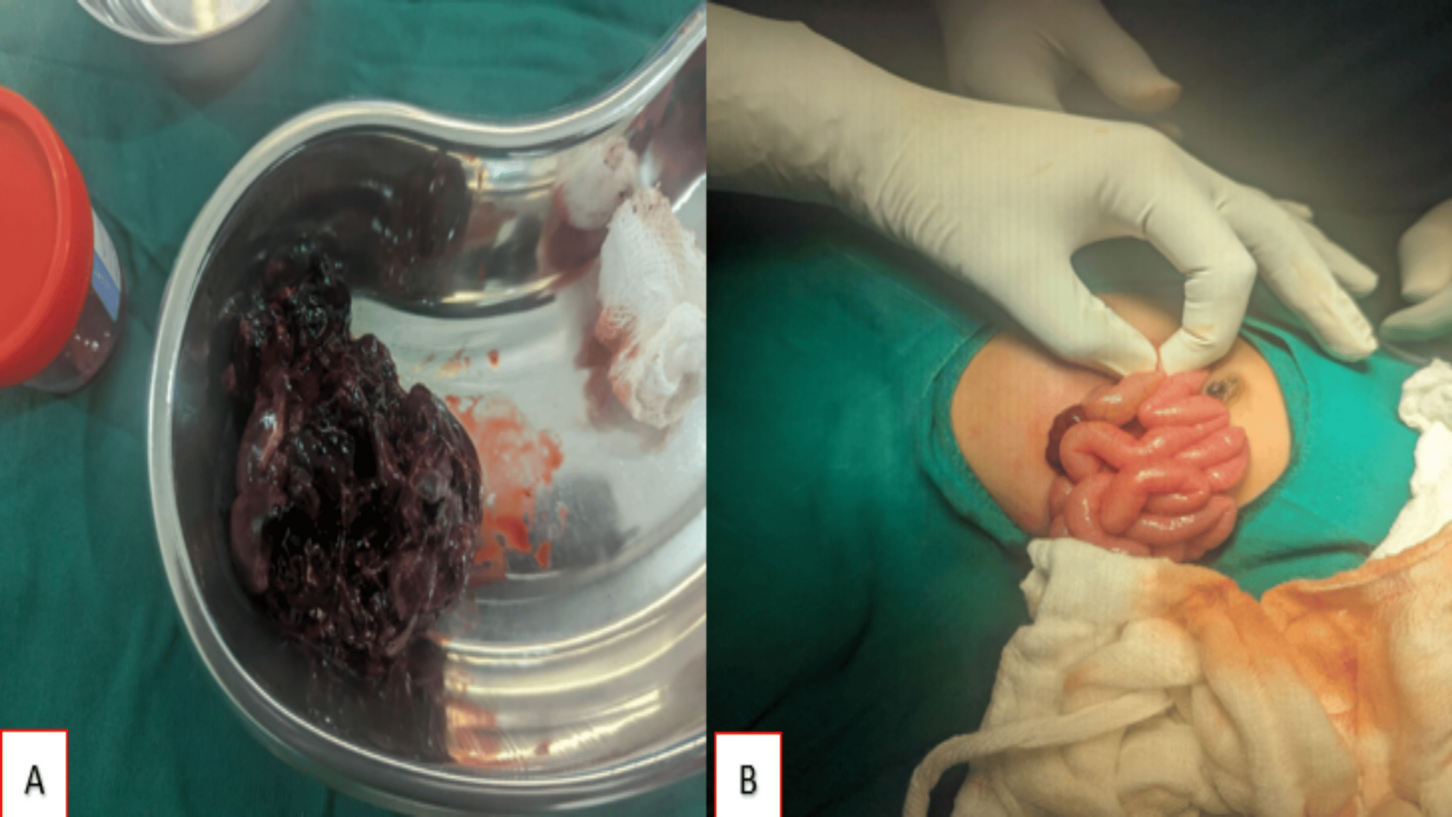
(A) Post-excision specimen showing a necrotic mass with haemorrhagic content. (B) Intact bowel loops.

The need for exploratory laparotomy was explained to the patient and family, and consent was obtained. The procedure began with a transverse incision to gain access to the abdominal cavity. Upon exploration, a large mass was discovered in the thoracoabdominal region (Figure 7A). The mass appeared necrotic and was situated in the retroperitoneal space. This mass was notably exerting pressure on and displacing adjacent vital organs, including the stomach, liver, colon, and spleen. Using bipolar cautery, the mass was meticulously dissected and successfully excised, minimizing damage to surrounding tissues. Intraoperatively, the bowel wall was examined and confirmed to be intact (Figure 7B). To manage any residual fluid accumulation and to monitor postoperative healing, a surgical drain was placed in the abdominal cavity before closure. The procedure was completed without immediate complications.

**Figure 8:**
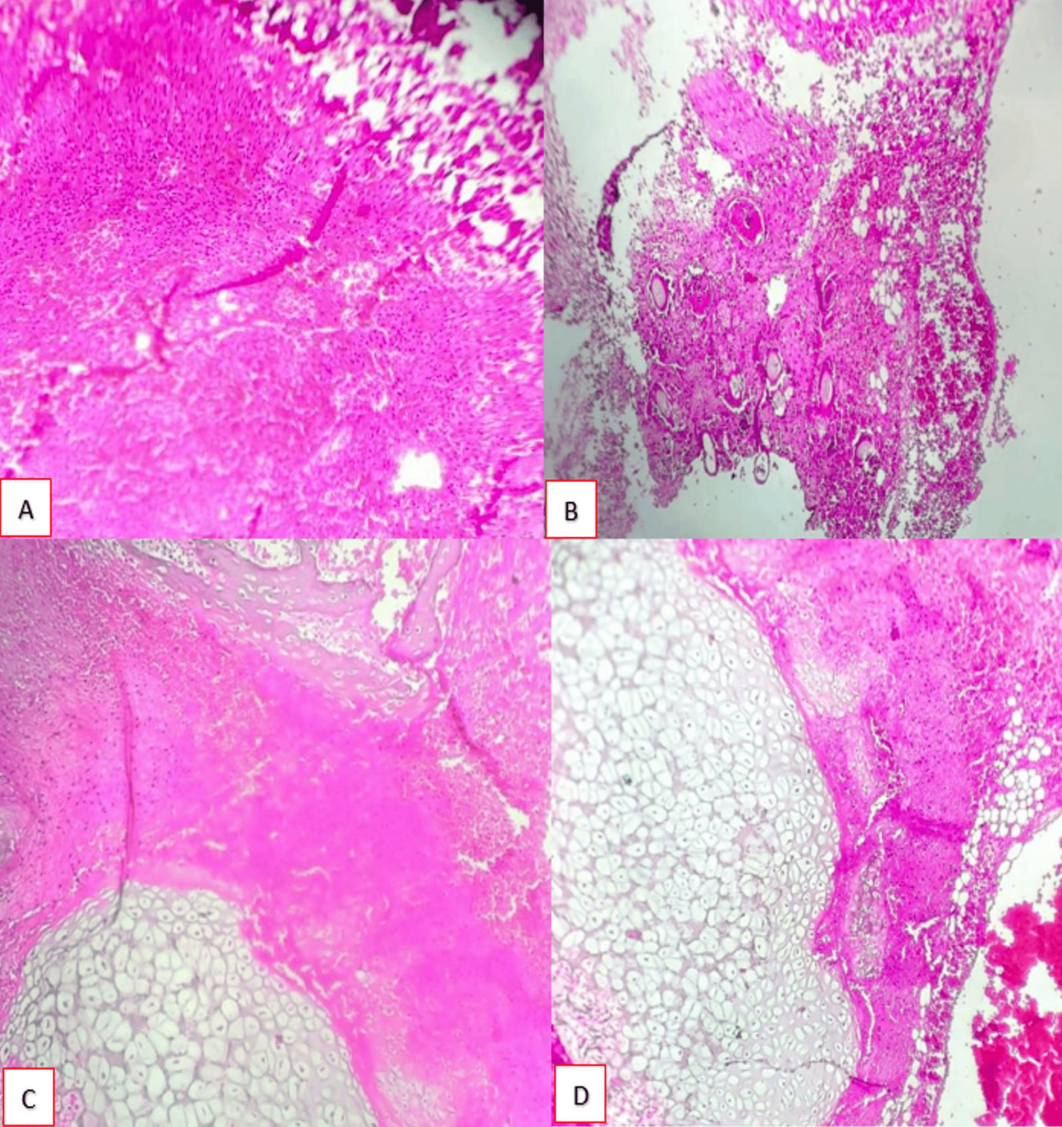
Histopathological findings. (A) Haemorrhage with infarctive changes (H&E; 10X); (B) Hair follicles in a haemorrhagic background (H&E; 10X); (C) Cartilage with bony tissue (H&E; 10X); (D) Cartilage with adjacent haemorrhage (H&E; 10X) Stain: hematoxylin and eosin (H%E); Magnification: 10X; Sample: Resected mass

The resected surgical specimen was sent for histopathological evaluation for further characterization, which revealed a tissue mass composed of extensive areas of necrosis and haemorrhage (Figure 8A) islands of hair follicles with adjacent haemorrhage (Figure 8B) and cartilage (Figures 8C, 8D) along with skin adnexal structures, indicating elements from more than one germ layer. The pathologist concluded that the features are suggestive of haemorrhagic infarction of a teratoma.

**Figure 9:**
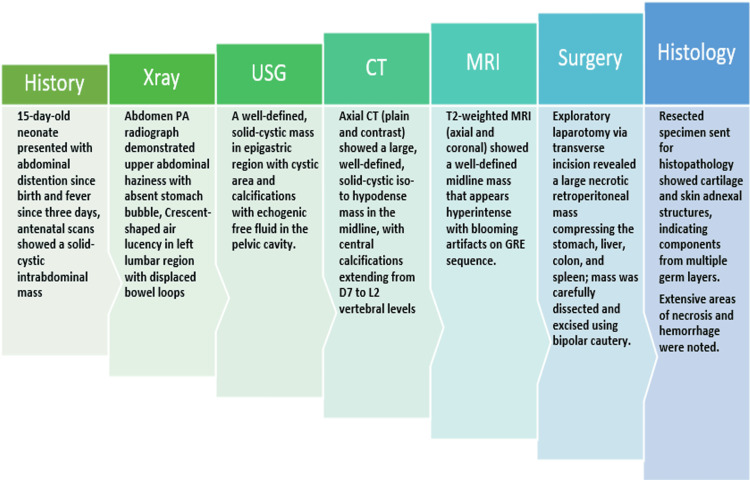
A concise timeline illustrating the sequence of events from clinical presentation to histopathological diagnosis. PA: posteroanterior; GRE: gradient-recalled echo

The concise timeline of the patient’s presentation to the final diagnosis is depicted in Figure 9. At the one-month postoperative follow-up, the neonate demonstrated favourable clinical progress with no signs of recurrence or complications. No follow-up laboratory tests were ordered for the patient. Serial sonographic evaluations confirmed the absence of residual or recurrent lesions, and the patient exhibited appropriate weight gain, normal feeding behaviour, and age-appropriate developmental milestones. A structured long-term surveillance protocol has been instituted. This includes periodic imaging and clinical evaluations at three-month intervals during the first postoperative year, with subsequent spacing based on clinical and radiologic stability.

## Discussion

Retroperitoneal teratomas in neonates consist of a group of rare congenital tumours. Its incidence is approximately 0.3% to 3% of all tumors and 1% to 10% of primary retroperitoneal tumors in children, far behind the ovarian and testicular locations [5]. These neoplasms arise from totipotent germ cells capable of differentiating into tissues from all three germ layers - ectoderm, mesoderm, and endoderm - resulting in a heterogeneous histologic composition that may include elements such as cartilage, neural tissue, epithelium, fat, and bone [6].

Retroperitoneal teratomas are often asymptomatic and may go unnoticed until they grow large enough to compress surrounding structures. When symptoms do occur, they typically include back or abdominal pain, nausea, vomiting, or constipation. On physical examination, common findings can include a palpable abdominal mass, tenderness, or progressive abdominal distension [1]. In our case, the patient presented to our department on the 15th day of life with a palpable abdominal mass and abdominal distension since birth.

Radiological imaging plays a central role in the detection, characterization, and surgical planning of retroperitoneal teratomas. In retroperitoneal teratoma, X-ray may demonstrate calcification (considered to be pathogenomic), which was not evident in the present case. Schey et al. have recommended only a plain abdominal X-ray and excision of the tumor if the characteristic calcification is demonstrated [7]. Lack et al. have also reported that the presence of bones or teeth on an X-ray was the most helpful in establishing a diagnosis [8].

Prenatal ultrasonography can occasionally detect these masses as complex, cystic-solid lesions in the abdomen. Postnatally, ultrasonography remains the first-line modality due to its accessibility and ability to differentiate between cystic and solid components. Ultrasonography shows an acoustic shadow, and occasionally, fat-fluid levels may be seen. Retroperitoneal teratomas can be predominantly cystic or completely solid in appearance [9]. Similar findings were noted in our case where the lesion appeared heterogeneous, predominantly hyperechoic with solid-cystic areas noted within.

Teratomas often appear as heterogeneous masses with echogenic areas corresponding to fat or calcifications. CT is particularly valuable in delineating the full extent of the mass, especially its relationship to adjacent structures and its internal composition - fat, fluid, soft tissue, and calcifications - which are hallmark imaging features of teratomas [10].

Plain radiographs help in differentiating calcified components of the teratoma, ultrasound can distinguish between cystic and solid components, and CT scan can differentiate between adipose tissue and bone masses [11]. However, the best modality that can provide better resolution of the soft tissues, precisely identify benign and malignant features and help in staging of the tumor is MRI. Other than their diagnostic roles, these radiologic modalities are of utmost importance in planning the surgical treatment [12].

There are multiple probable causes of large solid-cystic abdominal masses in neonates, and pinpointing the exact diagnosis can be challenging. These masses can resemble a variety of conditions, including congenital cysts of the pancreas, pancreatoblastomas, multi-cystic dysplastic kidneys, neuroblastoma, large Wilms' tumors, mesenteric cysts, or even lymphangiomas. Each of these has distinct features, but their appearances can often overlap, making careful evaluation essential [13].

Retroperitoneal germ cell tumors are usually quite large by the time they are discovered [13,14]. When a mass contains a mix of calcifications, fat, fluid, and soft tissue, it strongly points toward a teratoma, with calcifications commonly seen in most cases [14]. Neuroblastomas is one of the closest differentials which, in contrast, tends to encase blood vessels and may invade nearby organs [15]. Since both teratomas and neuroblastomas can appear as mixed masses with areas of calcification, distinguishing between them on imaging can sometimes be difficult.

Complete surgical excision is the mainstay of treatment for retroperitoneal teratomas. For mature teratomas, resection is typically curative. However, the close proximity of these tumors to vital vascular structures like the aorta and inferior vena cava can make surgical dissection challenging. Detailed preoperative imaging is therefore crucial for safe and complete resection. In some cases, particularly when haemorrhagic infarction has occurred, neonates may present acutely with abdominal distension, anaemia, or hemodynamic instability, requiring urgent surgical intervention [9].

The prognosis for neonates with completely resected mature retroperitoneal teratomas is excellent, with survival rates exceeding 90% [16]. Nevertheless, long-term follow-up is important, particularly in cases of immature or incompletely excised tumors, to monitor for recurrence or malignant transformation. Although mature teratomas are benign in nature, the patients must be closely monitored since malignant transformation occurs in 3-6% of the subjects [17]. Radiologic imaging continues to play a vital role in surveillance. Ultrasound evaluations are commonly scheduled at one, three, and six months after surgery, although more frequent imaging may be advised based on clinical suspicion of recurrence. In our case, no recurrence was noted at the six-month follow-up scan.

## Conclusions

Retroperitoneal teratomas, though rare in neonates, should be a key consideration in the evaluation of abdominal masses. Their distinct imaging features, particularly fat, calcifications, and solid-cystic composition, allow for accurate diagnosis and effective surgical planning. Radiology plays a vital role from detection to follow-up. With timely surgical excision, prognosis is excellent in most cases. A multimodal imaging approach remains essential for optimal management and early identification of potential complications.
